# Supplementary figures and images for: SARS-CoV-2-specific T cells generated for adoptive immunotherapy are capable of recognizing multiple SARS-CoV-2 variants
Source: PLoS Pathog. 2022 Feb 14;18(2):e1010339. doi: 10.1371/journal.ppat.1010339 (PMC8880869; doi:10.1371/journal.ppat.1010339)

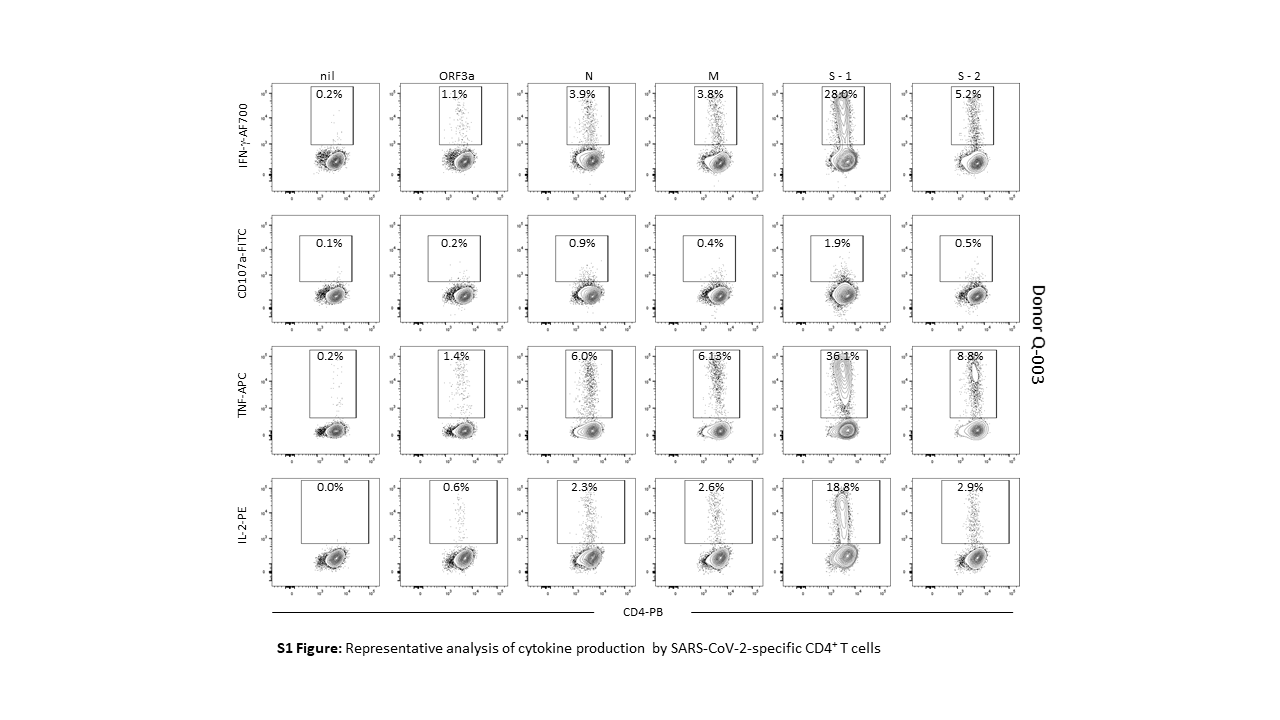

Supplement: S1 Fig — (TIF) [file ppat.1010339.s001.tif]

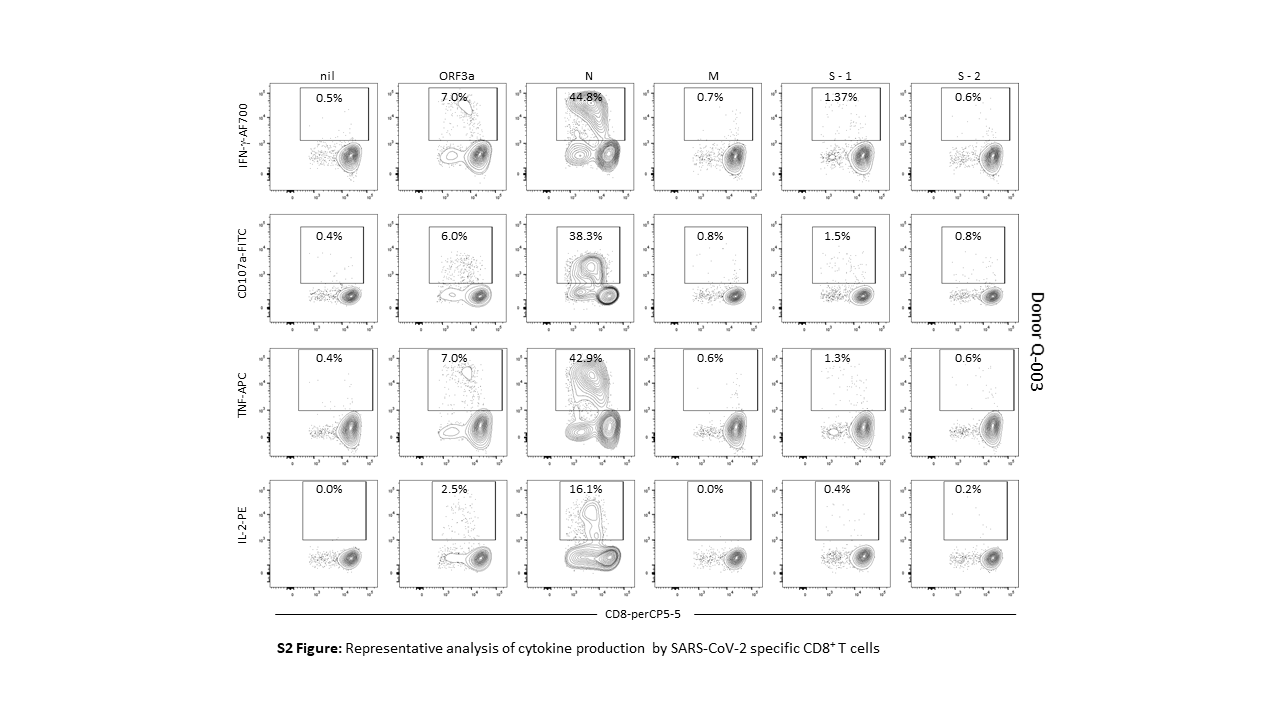

Supplement: S2 Fig — (TIF) [file ppat.1010339.s002.tif]

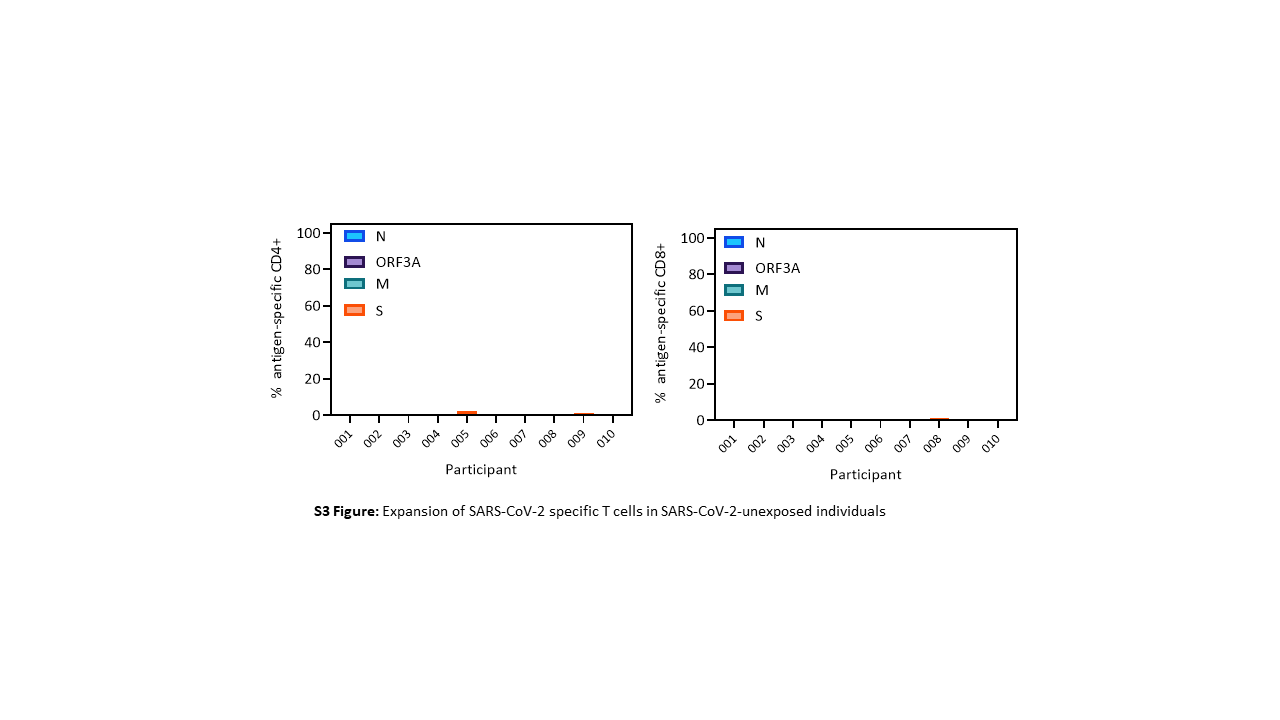

Supplement: S3 Fig — (TIF) [file ppat.1010339.s003.tif]

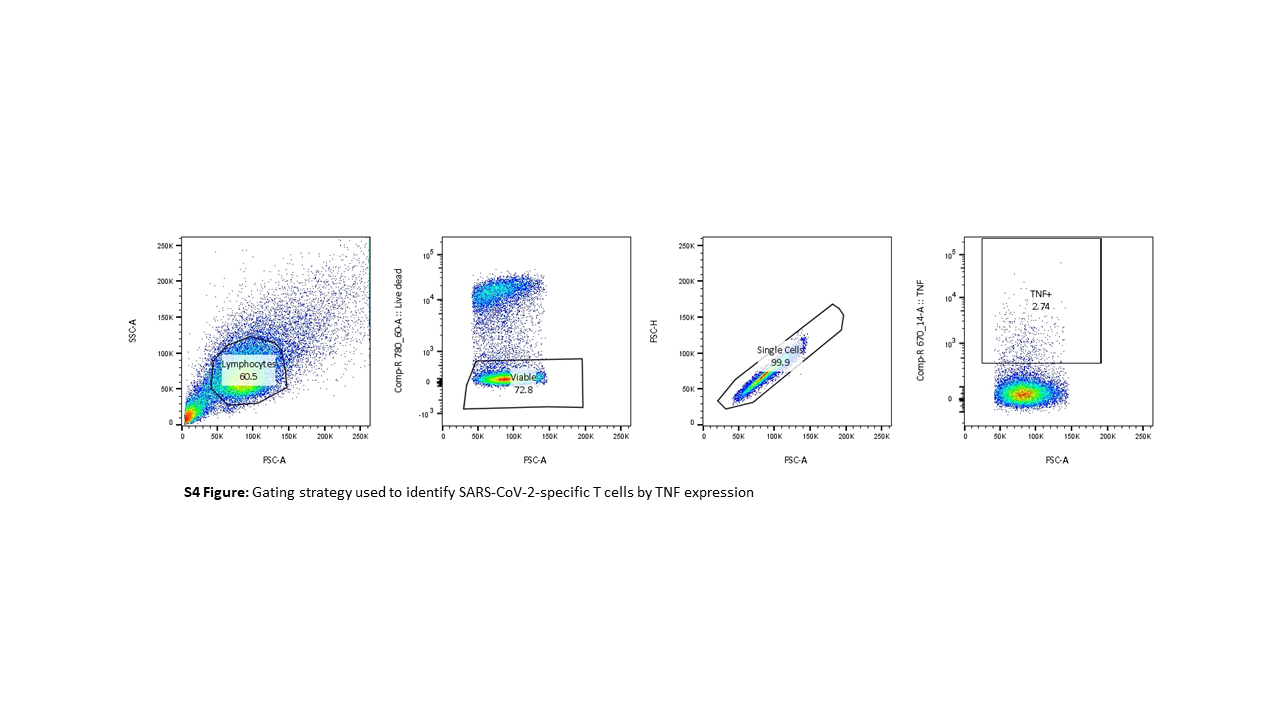

Supplement: S4 Fig — (TIF) [file ppat.1010339.s004.tif]

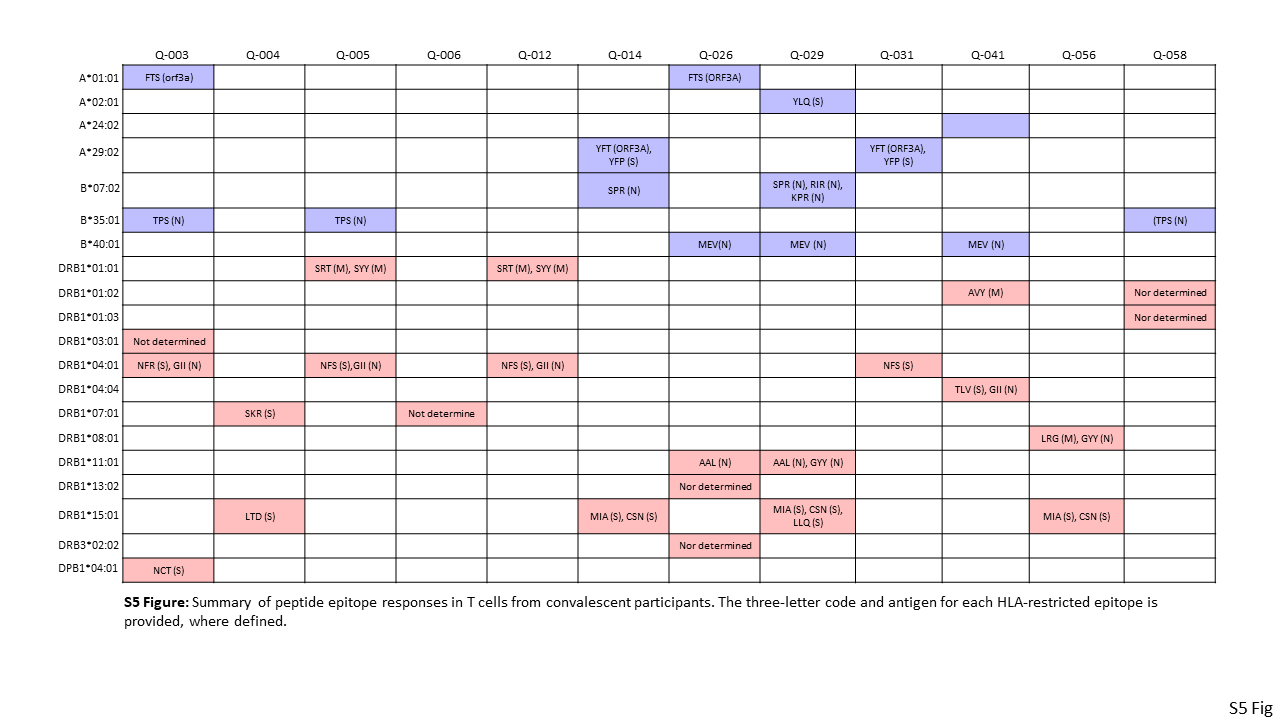

Supplement: S5 Fig — The three-letter code and antigen for each HLA-restricted epitope is provided, where defined. (TIF) [file ppat.1010339.s005.tif]

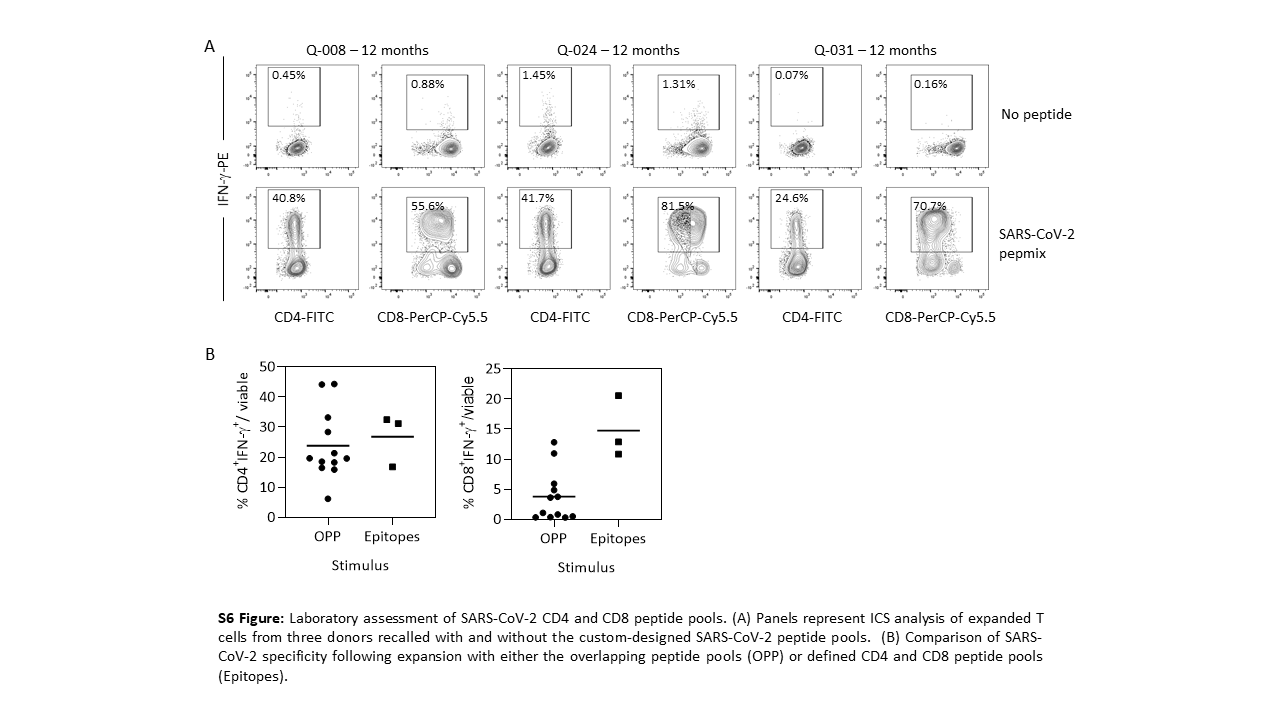

Supplement: S6 Fig — (A) Panels represent ICS analysis of expanded T cells from three donors recalled with and without the custom-designed SARS-CoV-2 peptide pools. (B) Comparison of SARS-CoV-2 specificity following expansion with either the overlapping peptide pools (OPP) or defined CD4 and CD8 peptide pools (Epitopes). (TIF) [file ppat.1010339.s006.tif]
