# Supplementary material for: SARS-CoV-2-specific T cells generated for adoptive immunotherapy are capable of recognizing multiple SARS-CoV-2 variants
Source: PLoS Pathog. 2022 Feb 14;18(2):e1010339. doi: 10.1371/journal.ppat.1010339 (PMC8880869; doi:10.1371/journal.ppat.1010339)
Supplement: S1 Table — (DOCX) [file ppat.1010339.s007.docx]

**S1 Table: Target cells used in the HLA-restriction assay**

| **HLA class I target cell lines** | **HLA class I alleles** | **Donor T cells** |
| --- | --- | --- |
| K562 | nil | All |
| K562-A0101 | A*01:01 | Q-003, Q-026 |
| K562-A0201 | A*02:01 | Q-012, Q-029, Q-031 |
| K562-A1101 | A*11:01 | Q-005, Q-058 |
| K562-A2402 | A*24:02 | Q-041 |
| K562-B0702 | B*07:02 | Q-004, Q-014, Q-029 |
| K562-B0801 | B*08:01 | Q-003 |
| K562-B3501 | B*35:01 | Q-003, Q-005, Q-058 |
| K562-B4001 | B*40:01 | Q-026, Q-028 |
| K562-B4402 | B*44:02 | Q-012 |
| K562-C0304 | C*03:04 | Q-029, Q-031 |
| K562-C0702 | C*07:02 | Q-029 |
| PHA-GR042 | A*11:01, A*29:02, B*35:01, B*58:01, C*04:01, C*07:18 | Q-014, Q-031 |
| PHA-024 | A*01:01, A*23:01, B*08:01, B*44:03, C*04:01, C*07:01 | Q-014, Q-031 |
| PHA-052 | A*24:02, A*26:01, B*15:01, B*15:17, C*03:03, C*07:01 | Q-031 |
| **HLA class II target cell lines** | **HLA class II alleles** | **Donor T cells** |
| T2 | nil | All |
| T2-DR1 | DRB1*01:01 | Q-005, Q-012 |
| T2-DR3 | DRB1*03:01 | Q-003 |
| T2-DR4 | DRB1*04:01 | Q-003, Q-005, Q-006, Q-012, Q-031 |
| T2-DR11 | DRB1*11:01 | Q-026, Q-029 |
| DAP-DR7 | DRB1*07:01 | Q-014, Q-031 |
| DUCAF LCL | DRB1*03:01, DRB3*02:02, DQB1*02:01, DPB1*02:02 | Q-003, Q-026 |
| SCHU LCL | DRB1*15:01, DRB5*01:01, DQB1*06:02, DPB1*04:02 | Q-004, Q-014, Q-029, Q-056 |
| LCL-033 | DRB1*04:04, DRB1*11:01, DQB1*03:01, DQB1*03:02 | Q-041 |
| LCL-035 | DRB1*01:02, DRB1*03:01, DQB1*05:01, DQB1*02:01 | Q-041, Q-058 |
| LCL-038 | DRB1*01:03, DRB1*04:04, DQB1*05:01, DQB1*03:02 | Q-058 |
| LCL-052 | DRB1*13:02, DRB1*14:04 | Q-026, Q-003 |
| LCL-058 | DRB1*07:01 DRB1*08:01, DQB1*02:02, DQB1*04:02 | Q-004, Q-006, Q-056 |
