# Supplementary material for: SARS-CoV-2-specific T cells generated for adoptive immunotherapy are capable of recognizing multiple SARS-CoV-2 variants
Source: PLoS Pathog. 2022 Feb 14;18(2):e1010339. doi: 10.1371/journal.ppat.1010339 (PMC8880869; doi:10.1371/journal.ppat.1010339)
Supplement: S2 Table — (DOCX) [file ppat.1010339.s008.docx]

**S2 Table: Summary of HLA-restriction analysis**

|  |  | MHC class I | | | | MHC class II | | | |
| --- | --- | --- | --- | --- | --- | --- | --- | --- | --- |
| Donor | Matched allele | ORF3a | N | M | S | ORF3a | N | M | S |
| Q-003 | Control | 0.00 | 0.00 |  |  |  | 0.00 | 0.00 | 0.00 |
|  | A*01:01 | 3.59 | 0.00 |  |  |  |  |  |  |
|  | B*08:01 | 0.00 | 0.00 |  |  |  |  |  |  |
|  | B*35:01 | 0.05 | 37.68 |  |  |  |  |  |  |
|  | DRB1*04:01 |  |  |  |  |  | 2.64 | 0.71 | 6.03 |
|  | DRB1*03:01 |  |  |  |  |  | 0.16 | 0.31 | 4.00 |
|  | DPB1*04:01 |  |  |  |  |  | 0.00 | 0.00 | 11.19 |
| Q-014 | Control | 0.00 | 0.00 |  | 0.00 |  |  |  |  |
|  | B*07:02 | 0.00 | 5.70 |  | 0.00 |  |  |  |  |
|  | A*29:02 | 2.46 | 0.05 |  | 1.65 |  |  |  |  |
|  | B*44:03 | 0.00 | 0.00 |  | 0.06 |  |  |  |  |
|  | DRB1*07:01 |  |  |  |  |  | 0.13 | 0.18 | 0.19 |
|  | DRB1*15:01 |  |  |  |  |  | 0.98 | 5.09 | 11.49 |
| Q-026 | Control | 0.24 | 0.00 |  |  |  | 0.00 | 0.03 | 0.00 |
|  | A*01:01 | 6.13 | 1.62 |  |  |  |  |  |  |
|  | B*40:01 | 0.61 | 7.28 |  |  |  |  |  |  |
|  | DRB1*11:01 |  |  |  |  |  | 2.80 | 14.96 | 1.65 |
|  | DRB1*13:02 |  |  |  |  |  | 1.84 | 1.20 | 2.24 |
|  | DRB3*02:02 |  |  |  |  |  | 0.04 | 0.17 | 1.12 |
| Q-031 | Control | 0.16 | 0.04 | 0.02 | 0.00 | 0.00 | 0.01 | 0.01 | 0.07 |
|  | A*29:02 | 4.92 | 0.19 | 0.18 | 27.02 |  |  |  |  |
|  | A*02:01 | 0.58 | 0.03 | 0.01 | 0.14 |  |  |  |  |
|  | B*15:01 | 0.12 | 0.74 |  |  |  |  |  |  |
|  | B*44:03 | 0.11 | 0.06 | 0.19 | 0.08 |  |  |  |  |
|  | C*03:04 | 0.12 | 0.06 |  |  |  |  |  |  |
|  | DRB1*04:01 |  |  |  |  | 0.44 | 5.94 | 1.02 | 11.77 |
|  | DRB1*07:01 |  |  |  |  | 0.05 | 0.08 | 0.12 | 0.26 |
| Q-029 | Control | 0.10 | 5.32 |  | 0.00 |  | 0.18 | 0.39 | 0.34 |
|  | A*02:01 | 6.12 | 1.16 |  | 0.14 |  |  |  |  |
|  | B*07:02 | 0.29 | 24.88 |  | 0.50 |  |  |  |  |
|  | B*40:01 | 0.46 | 15.68 |  | 0.03 |  |  |  |  |
|  | C*03:04 | 0.00 | 4.04 |  | 0.00 |  |  |  |  |
|  | C*07:02 | 0.00 | 2.65 |  | 0.00 |  |  |  |  |
|  | DRB1*11:01 |  |  |  |  |  | 8.44 | 7.53 | 2.54 |
|  | DRB1*15:01 |  |  |  |  |  | 4.29 | 3.02 | 18.42 |
| Q-012 | Control | 0.00 |  |  | 0.00 | 0.00 | 0.02 | 0.09 | 0.16 |
|  | A*02:01 | 0.27 |  |  | 0.25 |  |  |  |  |
|  | B*44:02 | 0.16 |  |  | 0.00 |  |  |  |  |
|  | DRB1*01:01 |  |  |  |  | 2.61 | 0.93 | 8.39 | 2.59 |
|  | DRB1*04:01 |  |  |  |  | 0.06 | 1.50 | 0.24 | 2.43 |
| Q-058 | Control |  | 0.11 | 0.19 |  |  | 0.24 | 0.54 | 0.66 |
|  | A*11:01 |  | 0.69 | 0.31 |  |  |  |  |  |
|  | B*35:01 |  | 0.57 | 2.45 |  |  |  |  |  |
|  | DRB1*01:02 |  |  |  |  |  | 1.32 | 7.09 | 1.78 |
|  | DRB1*01:03 |  |  |  |  |  | 1.99 | 2.28 | 1.30 |
| Q-005 | Control |  | 0.00 |  |  | 0.00 | 0.00 | 0.00 | 0.00 |
|  | A*11:01 |  | 0.28 |  |  |  |  |  |  |
|  | B*35:01 |  | 67.33 |  |  |  |  |  |  |
|  | DRB1*01:01 |  |  |  |  | 3.75 | 0.30 | 16.40 | 1.80 |
|  | DRB1*04:01 |  |  |  |  | 0.02 | 1.17 | 0.70 | 2.85 |
| Q-041 | Control | 0.00 | 2.60 |  |  |  | 0.00 | 0.00 | 0.00 |
|  | A*24:02 | 1.26 | 12.24 |  |  |  |  |  |  |
|  | B*40:01 | 0.55 | 45.16 |  |  |  |  |  |  |
|  | DRB1:01:02 |  |  |  |  |  | 1.32 | 39.58 | 6.28 |
|  | DRB1*04:04 |  |  |  |  |  | 2.49 | 3.80 | 8.70 |
| Q-004 | Control |  | 0.42 |  |  | 0.00 | 0.12 | 0.19 | 0.08 |
|  | B*07:02 |  | 0.27 |  |  |  |  |  |  |
|  | DRB1*07:01 |  |  |  |  | 2.01 | 1.78 | 1.28 | 5.25 |
|  | DRB1*15:01 |  |  |  |  | 0.38 | 1.19 | 1.65 | 21.09 |
| Q-006 | Control |  |  |  |  |  | 0.09 | 0.27 | 0.25 |
|  | DRB1*04:01 |  |  |  |  |  | 0.00 | 0.00 | 0.00 |
|  | DRB1*07:01 |  |  |  |  |  | 2.15 | 0.35 | 0.68 |
| Q-056 | Control |  |  |  |  | 0.00 | 0.00 | 0.00 | 0.00 |
|  | DRB1*08:01 |  |  |  |  | 1.63 | 3.30 | 2.83 | 0.99 |
|  | DRB1*15:01 |  |  |  |  | 0.00 | 2.66 | 1.84 | 16.52 |

Numbers represent the frequency of IFN-γ-producing CD8^+^ or CD4^+^ T cells

Highlighted boxes represent values 1% above control and deemed positive for HLA restriction
