# Supplementary material for: SARS-CoV-2-specific T cells generated for adoptive immunotherapy are capable of recognizing multiple SARS-CoV-2 variants
Source: PLoS Pathog. 2022 Feb 14;18(2):e1010339. doi: 10.1371/journal.ppat.1010339 (PMC8880869; doi:10.1371/journal.ppat.1010339)
Supplement: S3 Table — (DOCX) [file ppat.1010339.s009.docx]

**S3 Table**: SARS-CoV-2 CD8 Peptide Pool

| **Epitope** | **Antigen** | **HLA Restriction** |
| --- | --- | --- |
| QRNAPRITF | NCAP | B*27:05 |
| FPRGQGVPI |  | B*07:02 |
| RIRGGDGKM |  | B*07:02 |
| SPRWYFYYL |  | B*07:02 |
| ATEGALNTPK |  | A*11:01 |
| KPRQKRTAT |  | B*07:02 |
| AQFAPSASAF |  | B*15:01 |
| MEVTPSGTWL |  | B*40:01 |
| TPSGTWLTY |  | B*35:01 |
| KTFPPTEPK |  | A*03:01; A*11:01 |
| LLLDRLNQL |  | A*02:01 |
| FEYVSQPFL | Spike | B*49:01; B*40:01 |
| LEPLVDLPI |  | B*44:02/03 |
| LPIGINITRF |  | B*35:01 |
| YLQPRTFLL |  | A*02:01 |
| CVADYSVLY |  | A*29:02 |
| YFPLQSYGF |  | A*29:02 |
| LLQYGSFCTQ |  | Not Defined |
| SPRRARSVA |  | B*07:02 |
| IPFAMQMAY |  | B*35:01 |
| FTSDYYQLY | ORF3a | A*01:01 |
| YFTSDYYQLY |  | A*29:02 |
| LLYDANYFL |  | A*02:01 |
| WESGVKDCVVL |  | B*40:01 |
| KRWQLALSK |  | B*27:05 |
| RCSFYEDFLEY | ORF8 | B*44:03; B*18:04 |
| TTDPSFLGRY | ORF1ab | A*01:01 |
| KLWAQCVQL |  | A*02:01 |
| YAFEHIVY |  | B*35:01 |
| NVIPTITQMNL |  | A*02:05 |
| FVDGVPFVV |  | A*02:07 |
| LPYPDPSRI |  | B*51:01 |
