# Supplementary material for: SARS-CoV-2-specific T cells generated for adoptive immunotherapy are capable of recognizing multiple SARS-CoV-2 variants
Source: PLoS Pathog. 2022 Feb 14;18(2):e1010339. doi: 10.1371/journal.ppat.1010339 (PMC8880869; doi:10.1371/journal.ppat.1010339)
Supplement: S4 Table — (DOCX) [file ppat.1010339.s010.docx]

**S4 Table**: SARS-CoV-2 CD4 Peptide Pool

| **Epitope** | **Antigen** | **HLA Restriction** |
| --- | --- | --- |
| AIPTNFTISVTTEIL | Spike | Not Defined |
| CSNLLLQYGSFCTQL |  | DRB1*15:01 |
| GWTFGAGAALQIPFA |  | DRB1*01:01 |
| HWFVTQRNFYEPQII |  | DQB1*05:01 |
| INITRFQTLLALHRS |  | Not Defined |
| KLIANQFNSAIGKIQ |  | Not Defined |
| KSTNLVKNKCVNFNF |  | Not Defined |
| LLQYGSFCTQLNRAL |  | DRB1*15:01 |
| LREFVFKNIDGYFKI |  | Not Defined |
| LTDEMIAQYTSALLA |  | DRB1*15:01 |
| MIAQYTSALLAGTIT |  | DRB1*15:01 |
| NCTFEYVSQPFLMDL |  | DPB1*04:01 |
| NFRVQPTESIVRFPN |  | DRB1*04:01 |
| NFSQILPDPSKPSKR |  | DRB1*04:01 |
| PFFSNVTWFHAIHVS |  | Not Defined |
| PFGEVFNATRFASVY |  | Not Defined |
| PHGVVFLHVTYVPAQ |  | Not Defined |
| PINLVRDLPQGFSAL |  | DRB3*01:01 |
| PVAIHADQLTPTWRV |  | Not Defined |
| QALNTLVKQLSSNFG |  | Not Defined |
| RALTGIAVEQDKNTQ |  | Not Defined |
| SKRSFIEDLLFNKVT |  | DRB1*07:01 |
| TLEILDITPCSFGGV |  | Not Defined |
| TLVKQLSSNFGAISS |  | DRB1*04:04 |
| TRFASVYAWNRKRIS |  | Not Defined |
| VGGNYNYLYRLFRK |  | Not Defined |
| YFPLQSYGFQPTNGV |  | Not Defined |
| YNYLYRLFRKSNLKP |  | DPB1*02:01 |
| PHGVVFLHVTYVPAQ |  | Not Defined |
| AALALLLLDRLNQLE | NCAP | DRB1*03:01, DRB1*11:01, DRB1*13:01 |
| GIIWVATEGALNTPK |  | DRB1*04:01/04 |
| GYYRRATRRIRGGDG |  | DRB1*11:01, DRB1*08:01 |
| KPRQKRTATKAYNVT |  | DPB1*14:01 |
| KRTATKAYNVTQAFG |  | DPB1*14:01 |
| MKDLSPRWYFYYLGT |  | Not Defined |
| MSRIGMEVTPSGTWL |  | Not Defined |
| QELIRQGTDYKHWPQ |  | Not Defined |
| QVILLNKHIDAYKTF |  | Not Defined |
| SPRWYFYYLGTGPEA |  | Not Defined |
| TASWFTALTQHGKED |  | Not Defined |
| TKAYNVTQAFGRRGP |  | Not Defined |
| TWLTYTGAIKLDDKD |  | DRB1*07:01 |
| YFYYLGTGPEAGLPY |  | Not Defined |
| AVYRINWITGGIAIA | Membrane | DRB1*01:01/02 |
| ELVIGAVILRGHLRI |  | Not Defined |
| GLMWLSYFIASFRLF |  | Not Defined |
| LRGHLRIAGHHLGRC |  | DRB1*11:01/04; DRB1*08:01 |
| LRIAGHHLGRCDIKD |  | DRB1*11:01/04;  DRB1*08:01 |
| MWSFNPETNILLNVP |  | Not Defined |
| PKEITVATSRTLSYY |  | DRB1*01:01 |
| SRTLSYYKLGASQRV |  | DRB1*01:01; DRB1*07:01 |
| SYYKLGASQRVAGDS |  | DRB1*01:01; DRB1*07:01 |
