# Supplementary material for: SARS-CoV-2-specific T cells generated for adoptive immunotherapy are capable of recognizing multiple SARS-CoV-2 variants
Source: PLoS Pathog. 2022 Feb 14;18(2):e1010339. doi: 10.1371/journal.ppat.1010339 (PMC8880869; doi:10.1371/journal.ppat.1010339)
Supplement: Attachment — Submitted filename: Response to previous submission.docx [file ppat.1010339.s011.docx]

We thank the previous reviewers for their thoughtful comments on our previous submission. These have helped us make significant improvements to our manuscript. We have provided point-by-point descriptions of the responses below.

Reviewer #1: Major concerns.
**1. The title suggests that the T cells tested in the study were indeed a prepared therapeutic ready for use, and tested for efficacy in pre-clinical studies. However, the authors merely show that T cells responsive to SARS-CoV-2 antigen can be detected in a number of people who were previously infected. While they show that both CD4+ T cells and CD8+ T cells can be expanded ex-vivo, and that there appears to be a range of HLA-types represented, there is not much more presented. The point that T cells specific to SARS-CoV-2 are generated during natural infection and can be detected in convalescent donors through bulk level peptide antigen screening is expected and not novel. The authors stretch the message with the title to suggest that they therefore have a method ready for clinical application. This is a big jump, and as it currently stands, the title does not represent that data or the key findings at this point, and I feel is misleading. Extensive work would need to be completed before the authors could fully support the message of the title or add to some novelty in the field.**

We thank the reviewer for their insight and have made a number of significant changes to the manuscript to address this issue. Since the initial submission on the manuscript we have generated two clinical grade T cell therapy products in our GMP facility, Q-Gen Cell Therapeutics, at QIMR Berghofer Medical Research Institute. These products were generated using a pool of optimised peptide epitopes selected from our initial pre-clinical studies. Data on these clinical grade cell therapy products have now been included in a new Figure 6. Both these T cell products show potent reactivity against HLA matched SARS-CoV-2 epitopes from multiple variants of concern. -We hope this clearly demonstrates that we do in fact have clinical-grade T cell products available for administration to patients.

**2. The authors test antigen specificity using peptide pools. It would have been much more informative if actual epitopes were identified a the single peptide/MHC level rather than looking for random responses to a pool of hundreds of larger peptides. Knowing the true epitopes could then lead to the development of more directed T cell therapeutic strategies (maybe the development of CAR-T cell based approaches). This would require much more extensive analysis. The current proposal of using a bulk allogeneic prep of activated T cells off-covering a broad range of antigen specificities (most of which would not be known and would not even be SARS-CoV-2 specific) as an off-the-shelf strategy does not seem feasible, and at this stage may not be a safe or effective approach.**

We thank the reviewer for their helpful comments. We would like to firstly point out that the use of a bulk allogeneic prep of activated T cells is a fairly standard approach used by multiple groups worldwide to treat viral complications, particularly in immunosuppressed transplant patients. However, we do agree that the identification of defined peptide epitopes provides a better approach for adoptive therapy. The purpose of the original study using overlapping pools was to demonstrate the feasibility of generating broadly reactive SARS-CoV-2-specific T cells. Since completion of this work, we have identified the immunodominant epitopes targeted by the T cells in our expanded polyclonal populations. This is now provided in Supplementary Figure 5 and Table 2. As part of this analysis we have also provided a list of sequence variants present in SARS-CoV-2 variants of concern, including the recently emerged Omicron variant. We have now combined the epitopes defined in this study with other epitopes defined by our group and others to generate novel peptide pools (Supplementary Table S4) for our clinical-grade T-cell expansion outlined in Figure 6.

**3. Figure 2B suggests that there is a higher proportion of CD4+ T cells responsive to the virus than CD8+ T cells. However, the authors are using larger 15mer peptides that would skew the proportion of responsive cells toward MHC-class II presentation to activate CD4+ T cells more readily. It is likely that there are more CD8+ T cells generated than being recorded, and if the authors used 9mer peptides, the number of CD8+ T cells responsive would be more readily exposed. This again highlights a weakness of the paper and the work needed to dig down deep to measure the true level and antigen specificity of the responses, which truly would require assay readouts using single peptides (which otherwise could out compete each other for binding) and peptides of smaller more optimal size for MHC-1 loading to better detect CD8 specific responses.**

We agree with the reviewer that the expansion of CD8^+^ T cells is likely to be more efficient using defined epitopes. As a consequence, we have generated our new peptide pools based on the minimal CD8^+^ T cell epitopes. We demonstrate in a small qualification cohort (Supplementart Figure 6) who we assessed prior to clinical manufacture that the CD8^+^ T-cell response is improved using the minimal epitopes.

**Reviewer #2: One major issue is the absence of a negative control; how would T cells from a healthy donor who is SARA-CoV-2 naive respond to the stimulation with SARA-CoV-2 peptides? And what would be the % cytolysis? This would be essential to decide the level of background responses. Having an experiment comparing the responses from the selected subject (Q-029) and the other patient with high responses (Q-026) to a control could help clarify this point. Adding one patient from each of the other categories would even make it clearer.**

We thank the reviewer for the suggestion of negative controls of unexposed volunteers to assess SARS-CoV-2 specific T cells. While we have not used these in our cytolysis assays, data in Supplementary Figure 6 provides the T-cell responses from 10 unexposed individuals following expansion with overlapping sets of SARS-CoV-2 peptide pools, clearly demonstrating no activity against the SARS-CoV-2 peptide pools.

--------------------

**Reviewer #1: 1. In figure 2C, there are peptide induced responses that are not being included in the analysis because they are not producing IFNg. However they can be clearly seen based on down-regulation of CD8 expression. By marking the cells as being antigen specific based on the expression of IFNg only, the authors show that they are missing so much more information. That population of CD8 downregulated cells likely are producing other factors as pare of their specific effector function and should be recognized in the analysis.**

We thank the reviewer for this observation and agree that some of the activated T cells, particularly CD4^+^ T cells are generating cytokines other than IFN-γ. We have now modified the manuscript to show data representing the proportion of antigen-specific CD4^+^ or CD8^+^ T cells expressing any combination of cytokines (Figure 2). We have also now included calendar plots in this figure (Fig 2E&F) to clearly represent the cytokines produced by these cells.

**2. Figure D the results state, for CD4+ T cells, “only five demonstrated a response against ORF3a” when the figure shows that 11 have some response, at least 7 are obvious.**

We thank the reviewer for pointing out this error and have now corrected this. Please note we have updated this data to refer to responses >1% above background as our cut-off.

**3. Figure 3A&B: Labels on Y axis are missing and only show TNF, but labels should also represent cytokines IFNg, Il-2, CD107a.**

We have now removed Figure 3 and incorporated these data into Figure 2.

**4. Figure 4A: Labels are too small, colors chosen can be challenging for those with color blindness.**

We thank the reviewer for this comment and have now increased the font size and have tried to improve color choice.

**5. There is little indication regarding the number of cells generated using the techniques mentioned. If a bank of cells were to be used, there would need to be enough cells generated, cryopreserved, and viable once ready to use. Again, this may represent feasibility issues to cover the overarching message of the title.**

As part of our clinical expansion we have provided information on the fold expansion. In these two processes we have generated over 100 vials (4x10^7^ cells/vial) of SARS-CoV-2 specific T cells from each volunteer.

**6. Some of the methods and reagents used could be more clear. For example, "overlapping peptide pools" does not tell us much. I believe that these are 15mers overlapping by 11, and the number of peptides in the pools would be useful to know and would provide more transparency.**

We have now provided clarity in the methods on the peptide pools used.

**Reviewer #2: 1- It would be helpful if the authors discuss in the introduction the main criteria and desirable features for successful candidates for adoptive T-cell therapy against COVID-19. This could help the future reader navigate through the results section.**

We thank the reviewer for this comment; and have discussed some of the potentially desirable features of T cell therapy in the introduction.

**2- The authors did not provide an ample explanation for the specific markers and strategy utilized in Figure 4 for the phenotypic characterization of the cells.**

We have now provided more detail on these characteristics.

**3- Although the text mentions collective measures from all patients examined (e.g. the median), the main figures are displaying the individual patients. I would suggest moving these graphs to supplementary figures, and having collective figures showing the collective results in the main figures. Using scatter-bar plots could be a way to have the individual points still represented in the graphs.**

We thank the reviewer for this suggestion; however, our preference is to leave individual donors’ data in the main figures. This is a standard approach we use in cell therapy studies.

**4- Despite measuring several functions for the examined T cells, they do not show any representative flow cytometry plots of dual functions.**

As part of Supplementary Figures 1 and 2, we have now provided plots for each cytokine/function. Due to the assessment of more than two parameters, we use Boolean analysis rather than using dual function plots to determine if cells are displaying more than one function.

**Specific minor comments:
5- Figure 3: Panels A and B are missing the names of the measured cytokines/functions on top.
6- Page 3, line 18: “ … compared to chronic T-cell dysfunction observed in patients with severe COVID-19.” Does chronic accurately describe this condition?**

We have updated the text to correct these errors.

--------------------
