# Supplementary material for: SARS-CoV-2-specific T cells generated for adoptive immunotherapy are capable of recognizing multiple SARS-CoV-2 variants
Source: PLoS Pathog. 2022 Feb 14;18(2):e1010339. doi: 10.1371/journal.ppat.1010339 (PMC8880869; doi:10.1371/journal.ppat.1010339)
Supplement: Attachment — Submitted filename: Response to previous submission.docx [file ppat.1010339.s012.docx]

We thank the previous reviewers for their thoughtful comments on our previous submission.

**Please address the comments from Reviewer 1 regarding addition of latest references.**

*We thank the reviewer their suggested references and have added these into a new sentence in the discussion*
